# Supplementary material for: High microbiome and metabolome diversification in coexisting sponges with different bio-ecological traits
Source: Commun Biol. 2024 Apr 8;7:422. doi: 10.1038/s42003-024-06109-5 (PMC11001883; doi:10.1038/s42003-024-06109-5)
Supplement: Supplementary file 2 — Supplementary Information [file 42003_2024_6109_MOESM2_ESM.pdf]

Supplementary material

High microbiome and metabolome diversification in coexisting sponges with different bio-ecological traits

Valerio Mazzella, Antonio Dell'Anno, Néstor Etxebarría, Belén González-Gaya, Genoveffa Nuzzo, Angelo Fontana, Laura Núñez-Pons

Supplementary material contains:
Supplementary materials and methods
Supplementary figures S1-S6
Supplementary Tables S1-S12
Supplementary references

Supplementary Material and Methods

1.0. Sample collection and processing

Sponge samples collected at the three diffrent sites were divided in sub-samples and properly stored for downstream analyses as follows:

- For microscope observations ~0.5 cm^3 pieces were fixed in 2.5% glutaraldehyde.
- For microbiome characterization ~0.5 cm^3 pieces were cut with sterile scalpel, rinsed with sterile seawater, snap frozen in liquid nitrogen and stored at -80 °C.
- For metabolomics analysis ~10 cm^3 sub-samples were stored at -80 °C. For these analyses were selected only samples from the sites GF and SA.

From each sampling site seawater samples (10 L) were also collected in triplicate. Seawater (1 L) was filtered through 0.22 μm pore size filters (Millipore MF^{TM}-Membrane) and stored at -80 °C.

1.1. Transmission electron microscope (TEM) observations

Samples were washed three times with filtered seawater, incubated with 1% osmium tetroxide for 1 hour and rinsed 4 times with filtered seawater. Four dehydration steps were performed for 30 minutes each with increasing acetone concentrations: 50%, 70%, 80% and 90%. Samples were then placed in 100% acetone for 30 minutes three times. Samples were embedded in Spurr resin on the shaker plate: two hours 1/3 Spurr resin / Acetone; three hours 2/3 Spurr resin / acetone; overnight 3/1 Spurr resin / acetone; two hours 100% Spurr resin; overnight in 100% Spurr resin at 60 °C for 24-48 h.

1.2. DNA extraction, amplification and sequencing

Quantity and quality of the extracted DNA from sponges and seawater filters were determined using a Thermo Scientific Nanodrop™ 1000. DNA aliquots were sent to Personal Genomics laboratories (www.personalgenomics.it) for amplicon library construction preparations and sequencing. For bacterial and archaeal diversity analysis, the V3-V4 hypervariable region of the 16S rRNA gene (Escherichia coli position: 341-805) was amplified using the bacterial and archaea universal primers: Bakt_341F 5′-CCTACGGGNGGCWGCAG-3′, Bakt_805R 5′-GACTACHVGGGTATCTAATCC-3′ [1] following the Illumina protocol.Amplification reactions were prepared using 2.5 μL DNA (5 ng/μL), 5μL forward primer (1 μM), 5 μL reverse primer (1 μM) and 12.5 μL KAPA HiFi HotStart ReadyMix, for a total volume of reaction of 25 μL. PCR were performed in a Veriti™ 96-Well Thermal Cycler using the following thermocycling conditions: 95°C for 3 minutes followed by 25 cycles of 95°C for 30 seconds, 55°C for 30 seconds and 72°C for 30 seconds and a final elongation

performed at 72°C for 5 minutes. After purification with AMPure XP beads the amplicons were analysed by a MiSeq sequencer (Illumina platform).

1.3. Quality control and filtering of raw sequence data

Reference sequences were trimmed to span the query V_3-V_4 region (~411 bp). ASV feature tables were further filtered considering a minimum of 7 reads throughout all samples, and removing untargeted features, such as “Chloroplast” or “Mitochondria”. Alpha rarefaction curves were build setting the lowest number of reads to 7038. One sample belonging to the sponge species *C. crambe* , one belonging to the sponge *P. ficiformis* and one seawater sample were dropped after rarefaction, due to sequencing depth not passing the established threshold of minimal reads.

1.4. Metabolomics profiling

Chemical profile screenings were performed by thin layer chromatography (TLC) using three elution conditions: $CHCl_3$/MeOH 9:1, EP/EE 6:4 and $CHCl_3$/MeOH/H2O 65:25:4.

Dry extracts were dissolved in 4 mL of MeOH:$CHCl_3$ (1:1), and an aliquot was diluted 40 times in MeOH:iPrOH (1:1). Then 10 µL were injected in a Thermo Scientific Dionex UltiMate 3000 UHPLC coupled to a Thermo Scientific Q Exactive focus quadrupole-Orbitrap mass spectrometer, equipped with a heated electrospray ionization (HESI) source (Thermo, CA, USA). Chromatographic separations were performed through a Kinetex^R Biphenyl (2.6 µm, 100 A, 100 × 2.1 mm) column (Phenomenex, USA) using a solution containing 5 mM ammonium formate and 0.1% formic acid) and methanol as mobile phases. LC multistep gradient flux was kept constant at 0.3 mL min^{-1} starting with 40% organic mobile phase, increasing until 80% in 2 minutes and reaching 100% at minute 15. It was maintained for 7 min. and it returned to initial conditions over 5 min’ delay. Column was kept at 35°C and samples were maintained at constant temperature (4°C) in the auto sampler. Samples were analyzed on positive and negative mode in the full scan – data dependent MS2 (Full MS-ddMS2) discovery acquisition mode. For the Full MS, resolution was set at 70,000 (tolerance of 5 ppm), with a scan range of 150 to 18000 *m/z*; and for ddMS2, resolution was diminished to 17,500 with switching normalized collision energies of 10, 20 and 40 eV. One microscan per cycle was allowed in the Full MS, with an AGC target 1×10^6 and a maximum IT of 100 ms. At the ddMS2 the isolation window was of 0.8 m/z, with and automatic gain control (AGC) target of 2×10^5, with a maximum intensity threshold of 75 milliseconds, over a three look counts. Dynamic exclusion was set as automatic and no other thresholds of apex trigger or exclusion were used. HESI source temperatures were set for the capillary at 320° C and for the auxiliary gas heater at 350° C, and spray voltage was set at 3.20 kV. Source gases fluxes were fixed at 60 arbitrary units (au) for the sheath gas, 35 au for the auxiliary

gas and 1 au for the sweep gas. S-lenses RF level was set at 55.0. Calibration of the instrument was conducted both for negative and positive ionization modes prior to analysis using Pierce LTQ ESI Calibration Solutions (Thermo Scientific, Waltham, Massachusetts, United States).
The instrument was controlled with the software Xcalibur 3.1 (Thermo). A calibration curve was prepared injecting standards of 21 lipids, in five calibration solutions (Cal01 to Cal05) with increasing concentrations (~0.001 to ~3.585 µg/µl), injected before the samples.

1.5. Metabolomics data analysis

LipidSearch v4.0 (Thermo Fisher Scientific) was used to process the UHPLC-q-Orbitrap data in non-targeted mode, to identify and plot metabolomic features grouped as molecule class profiles. PERMANOVA was used to detect significant differences between the sponge species. Pairwise Adonis was used to perform multilevel comparisons (“vegan” R package) [2]. Networks of single compounds and molecular classes across sponge species were visualized and computed by Gephi v0.9.2 [3]. Similarity percentage analysis (SIMPER) [4] was run on PRIMER 6+ software [5].

Supplementary figures

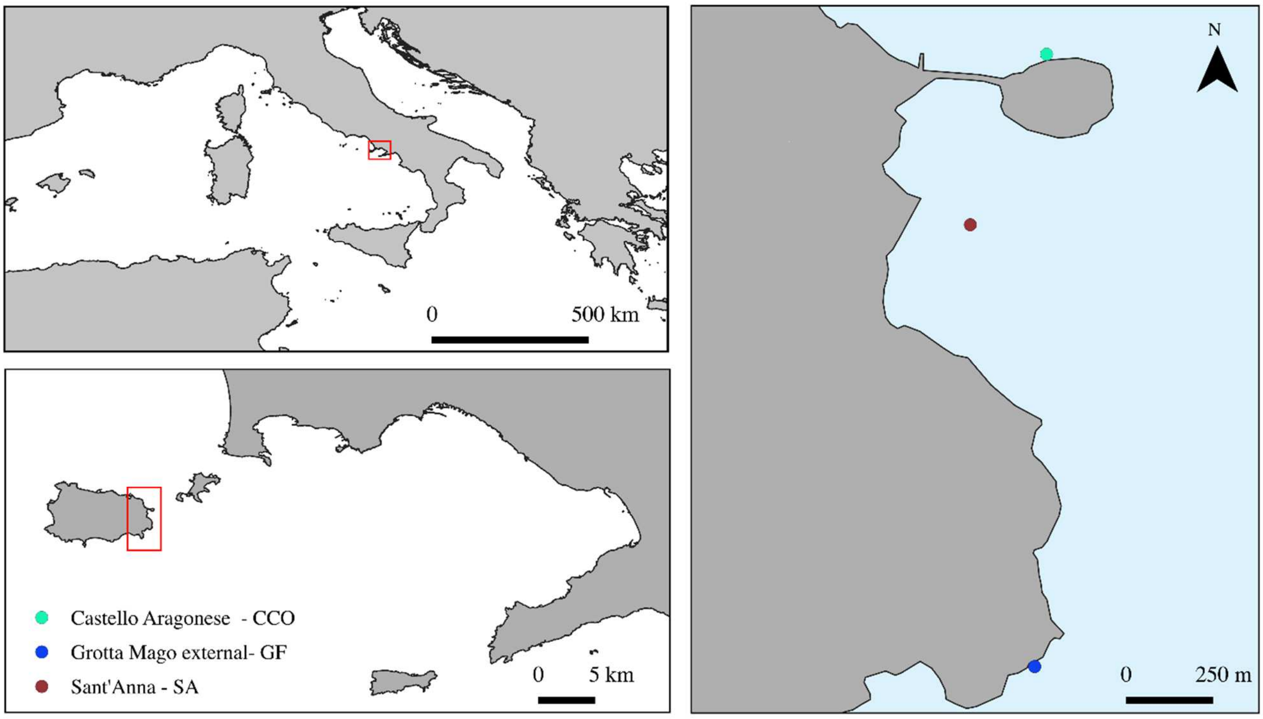

Figure S1. Map of the study area (Island of Ischia, southern Tyrrhenian Sea) showing the three sampling sites: Castello Aragonese (CCO), Sant'Anna (SA), Grotta Mago external (GF). Figure S1 was created using the Open Source QGIS software (QGIS Development Team, v3.28). The shapefiles were downloaded by the open access Italian National Institute for Statistics (Istat) website:
https://www.istat.it/storage/cartografia/confini_amministrativi/generalizzati/2023/Limiti01012023_g.zip

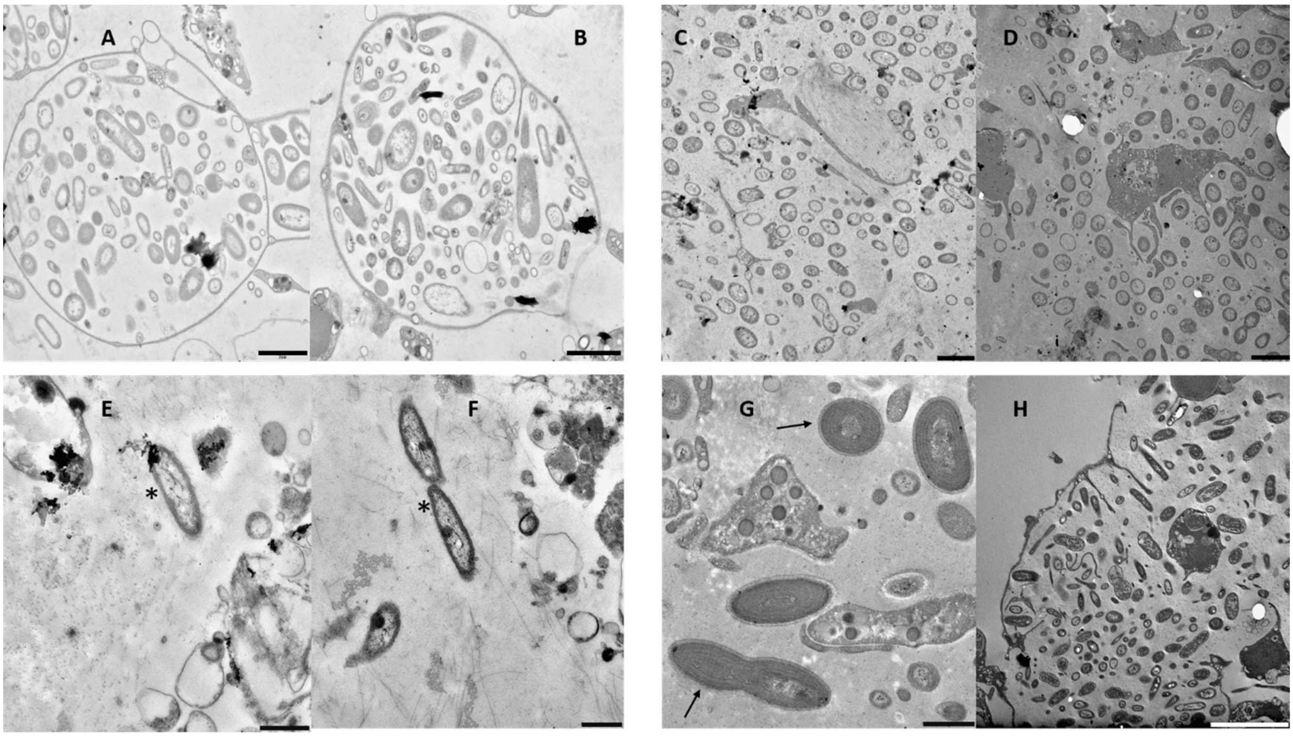

Figure S2. Transmission electron microscopy images of the four sponge species. A-B) Bacteriocytes in specimens of the sponge *P. ficiformis*. C-D) Bacteria in the sponge *C. reniformis*. E-F) Free bacteria in the mesohyl of *C. crambe* (black asterisks * indicate bacteria). G) Cyanobacteria in the bacteriocytes of the sponge *C. nucula.* (black arrows indicate a cyanobacterium on the top, and two dividing cyanobacteria on the bottom of the picture). H) Details of a bacteriocyte in the sponge *C. nucula.* Scalebars: A-B-C-D: 2 µM; E-F: 0.5 µM; G: 1 µM; H: 5 µM.

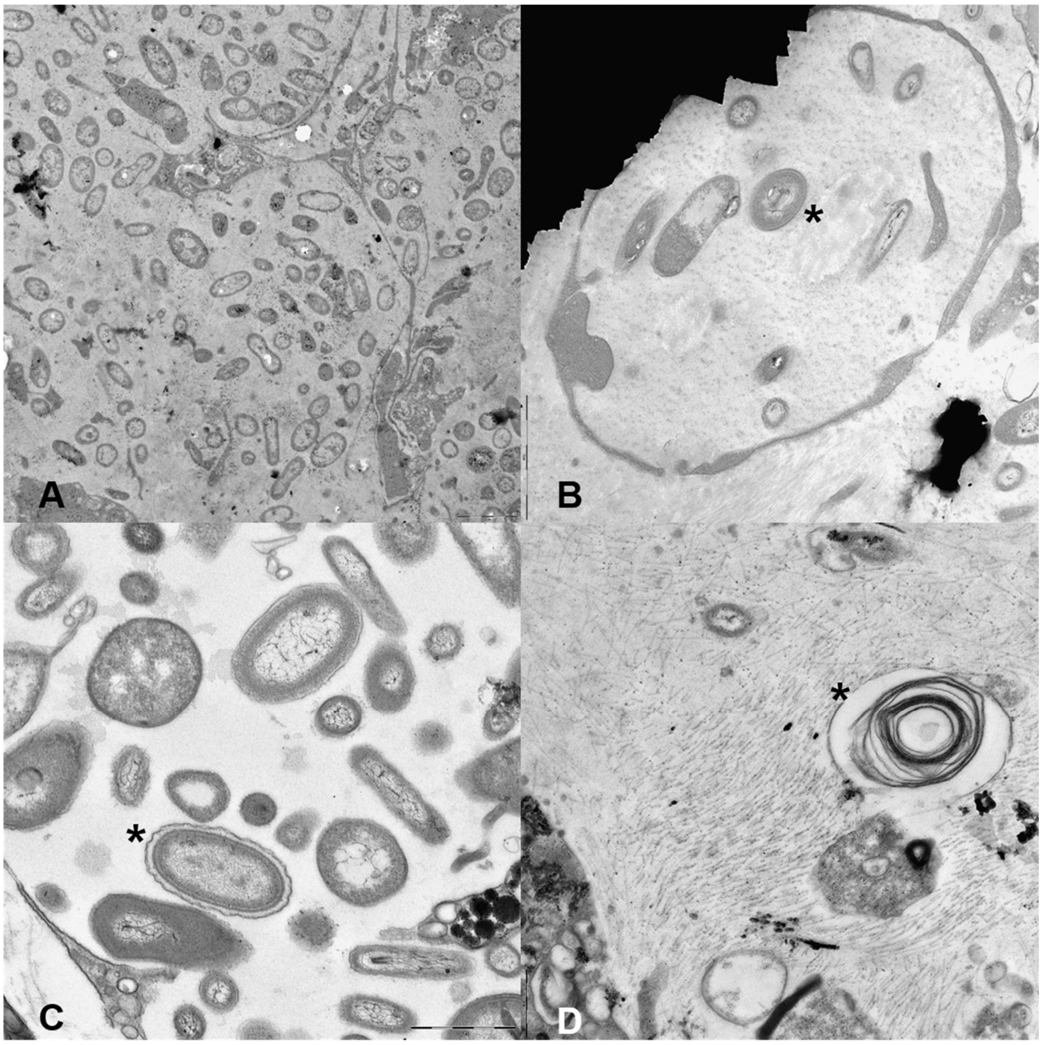

Figure S3. Supplementary images of the transmission electron microscopy (TEM) analysis. A) Microbes in the sponge *Chondrosia reniformis* B) Bacteriocyte-like structure found in the sponge *Chondrilla nucula* (B). C) Details on microbes found in the sponge *Petrosia ficiformis*. D) Cyanobacterium-like cell in the mesohyl of the sponge *Crambe crambe*. Asterisks (*) in the images B-C-D highlights morphologically different cyanobacterial-like cells in the sponges *C. nucula* (B), *P. ficiformis* (C) and *C. crambe* (D).

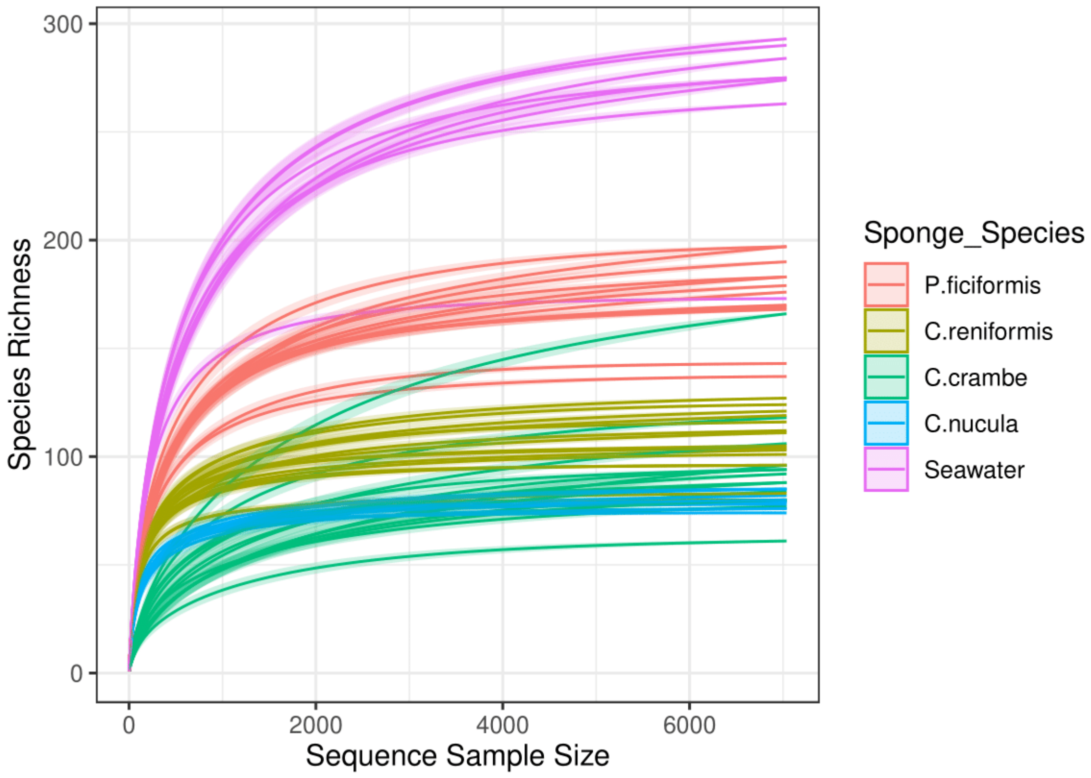

Figure S4. Rarefaction curves for the entire dataset comprising the four target sponge species *Petrosia ficiformis, Chondrosia reniformis, Crambe crambe*, and *Chondrilla nucula*. Each species is represented by different colour curves and each curve represents a sample. Sequences were rarefied to 7800 for traditional diversity analyses, in order to capture the maximum diversity at which deeper sequencing would not significantly acquire new taxa, while losing the lowest amount of samples. All of the samples reached a plateau within this threshold.

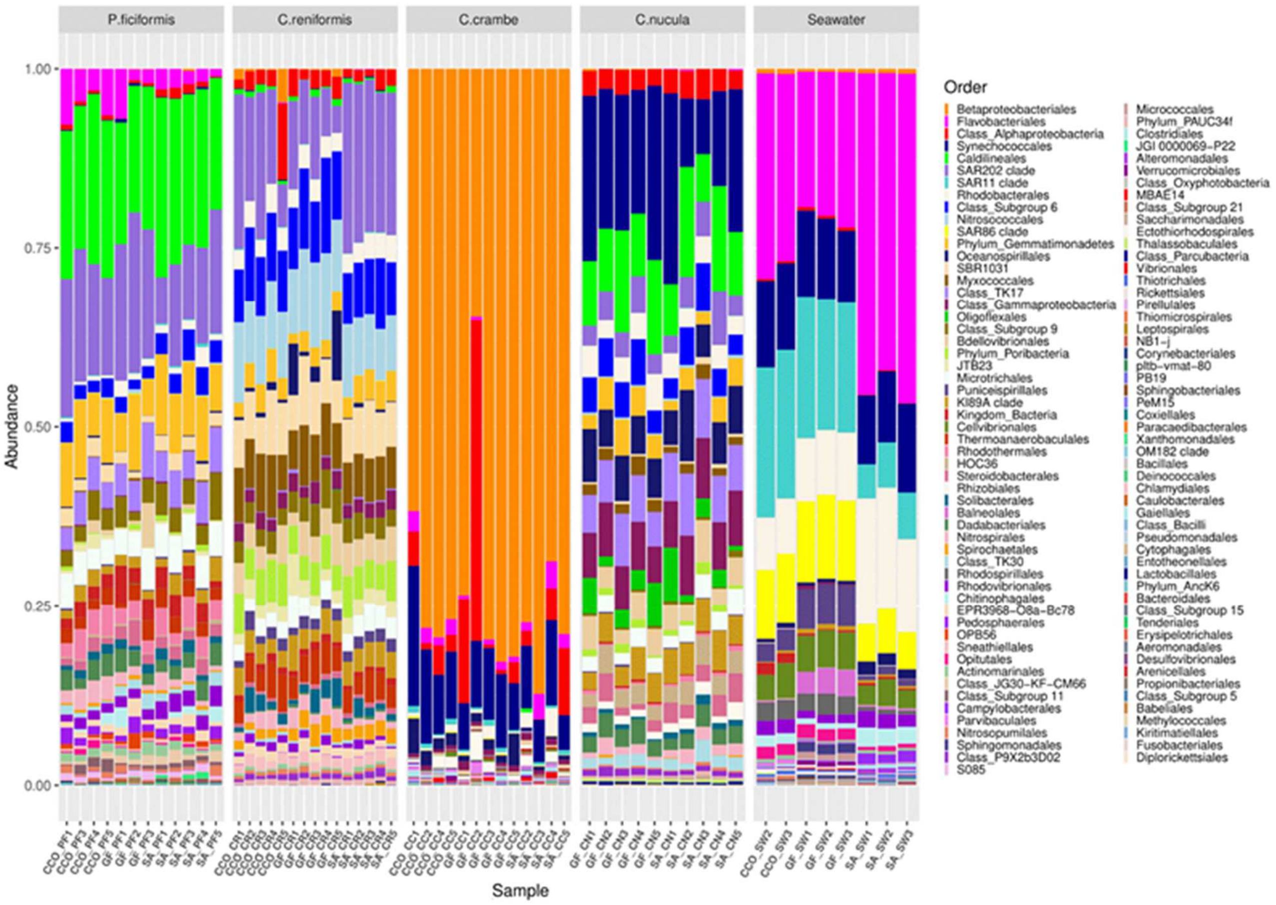

Figure S5. Supplementary taxa barplot reporting all the microbiomes of the four sponge species involved in this study. The low microbial abundance *C. crambe* was dominated by one bacterial strain while all the other three high microbial abundance sponges *P. ficiformis*, *C. reniformis*, *C. nucula* showed a high bacterial diversity. All the sponge associated microbiome were different from the microbial community present in the surrounding seawater.

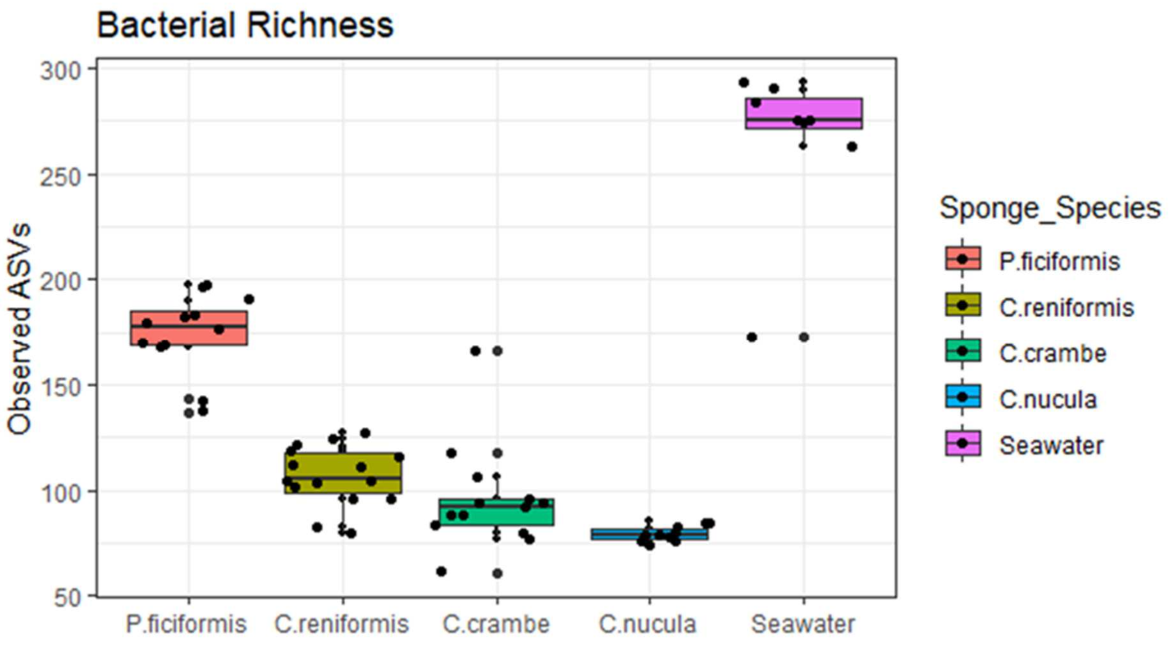

Figure S6. Alpha diversity boxplots of the observed ASVS found in the four sponge species involved in this study.

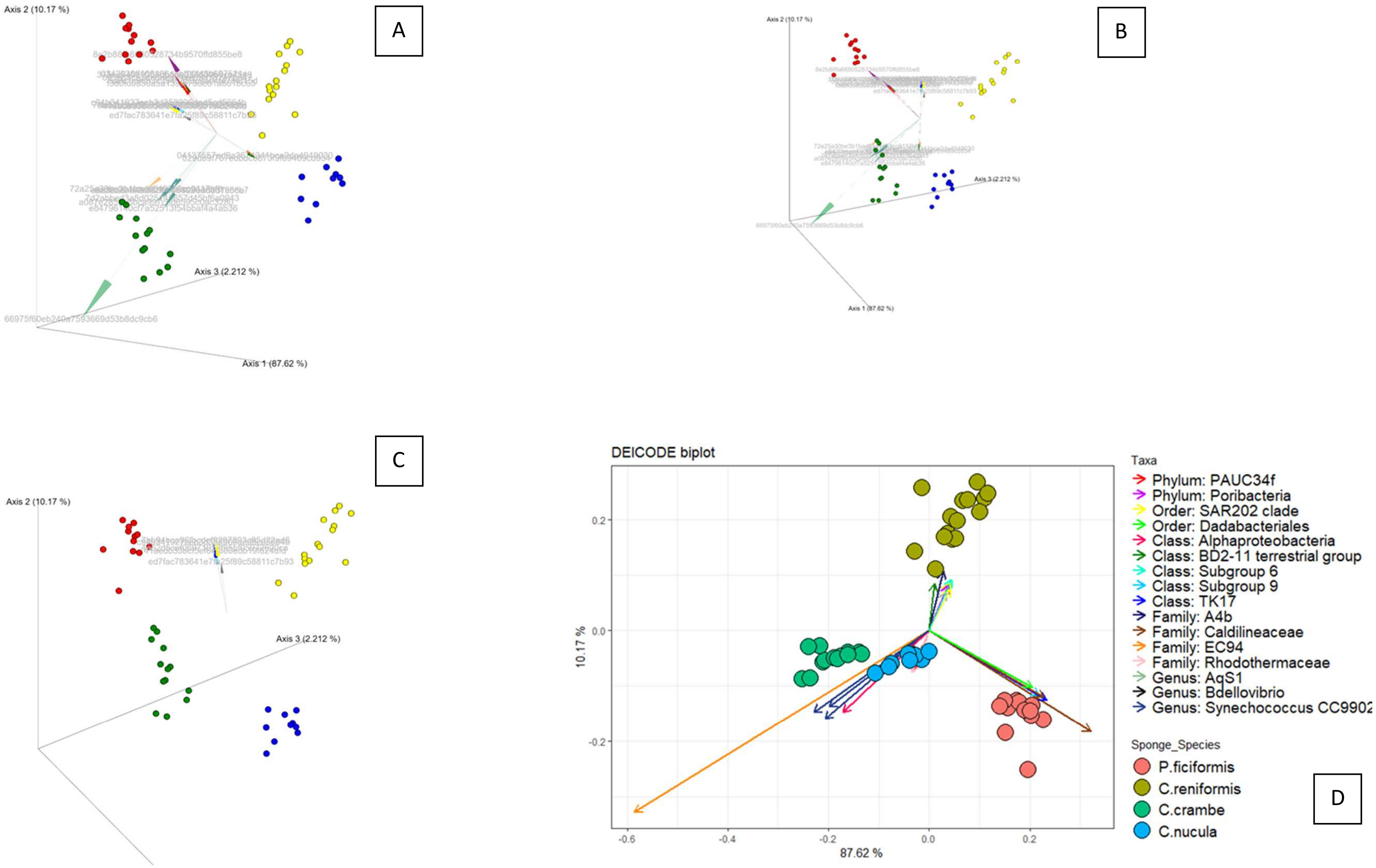

Figure S7. A-B-C) Three dimensional representation of the compositional RPCA biplot represented in Fig. 4 (main text). D) Compositional RPCA biplot (PC2 and PC3 of the Fig.4 in the main text) of beta diversity of bacterial communities associated with the four sponge species based on Aitchison distance .

Table S1. Metadata table resuming the principal informations of each sample. SampleID refers to the label of each sample obtained combining the name of the sampling site and the name of the relative sponge species plus a unique identifier number of the replicate. Sampling site refers to the code of each sampling location: CCO (Castello Aragonese), GF (Grotta Mago external), SA (Sant'Anna). Sponge species refers to the contracted scientific name of each sponge species*: Crambe crambe*, *Petrosia ficiformis*, *Chondrosia reniformis* and *Chondrilla nucula*. *All samples in the same species are of the same phenotype: pigmented and massive for *P. ficiformis* and *C. reniformis*, pigmented and lobular for *C. nucula* and encrusting for *C. crambe*, and all from illuminated habitats and analogous conditions.*"*

SampleID	Sampling Site	Sponge_Species
CCO_CC1	CCO	C.crambe
CCO_CC2	CCO	C.crambe
CCO_CC4	CCO	C.crambe
CCO_CC5	CCO	C.crambe
CCO_CR1	CCO	C.reniformis
CCO_CR2	CCO	C.reniformis
CCO_CR3	CCO	C.reniformis
CCO_CR4	CCO	C.reniformis
CCO_CR5	CCO	C.reniformis
CCO_PF1	CCO	P.ficiformis
CCO_PF3	CCO	P.ficiformis
CCO_PF4	CCO	P.ficiformis
CCO_PF5	CCO	P.ficiformis
CCO_SW2	CCO	Seawater
CCO_SW3	CCO	Seawater
GF_CC1	GF	C.crambe
GF_CC2	GF	C.crambe
GF_CC3	GF	C.crambe
GF_CC4	GF	C.crambe
GF_CC5	GF	C.crambe
GF_CN1	GF	C.nucula
GF_CN2	GF	C.nucula
GF_CN3	GF	C.nucula
GF_CN4	GF	C.nucula
GF_CN5	GF	C.nucula
GF_CR1	GF	C.reniformis
GF_CR2	GF	C.reniformis
GF_CR3	GF	C.reniformis
GF_CR4	GF	C.reniformis
GF_CR5	GF	C.reniformis
GF_PF1	GF	P.ficiformis
GF_PF2	GF	P.ficiformis
GF_PF3	GF	P.ficiformis
GF_SW1	GF	Seawater
GF_SW2	GF	Seawater
GF_SW3	GF	Seawater
SA_CC2	SA	C.crambe

SA_CC3	SA	C.crambe
SA_CC4	SA	C.crambe
SA_CC5	SA	C.crambe
SA_CN1	SA	C.nucula
SA_CN2	SA	C.nucula
SA_CN3	SA	C.nucula
SA_CN4	SA	C.nucula
SA_CN5	SA	C.nucula
SA_CR1	SA	C.reniformis
SA_CR2	SA	C.reniformis
SA_CR3	SA	C.reniformis
SA_CR4	SA	C.reniformis
SA_CR5	SA	C.reniformis
SA_PF1	SA	P.ficiformis
SA_PF2	SA	P.ficiformis
SA_PF3	SA	P.ficiformis
SA_PF4	SA	P.ficiformis
SA_PF5	SA	P.ficiformis
SA_SW1	SA	Seawater
SA_SW2	SA	Seawater
SA_SW3	SA	Seawater

Table S2. Output of the Kruskal-Wallis analysis carried out on Shannon index data. P-value threshold: 0.05.

```
Kruskal-Wallis rank sum test: shannon index by sponge species

Kruskal-Wallis chi-squared = 54.163, df = 4, p-value = 4.866e-11

Pairwise comparisons using Wilcoxon rank sum test:

             C.crambe C.nucula C.reniformis P.ficiformis
C.nucula     3.5e-06  -        -            -
C.reniformis 5.3e-07  2.0e-06  -            -
P.ficiformis 1.9e-06  5.2e-06  2.0e-06      -
Seawater     1.2e-05  4.6e-05  5.8e-06      1.8e-05
```

Table S3. Output of the Kruskal-Wallis analysis carried out on the number of observed ASVs. P-value threshold: 0.05.

```
Kruskal-Wallis chi-squared = 47.167, df = 4, p-value = 1.407e-09

Pairwise comparisons using Wilcoxon rank sum test:

             C.crambe C.nucula C.reniformis P.ficiformis
C.nucula     0.01010  -        -            -
C.reniformis 0.02851  0.00026  -            -
P.ficiformis 0.00020  0.00026  0.00012      -
Seawater     0.00031  0.00062  0.00026      0.00220
```

Table S4. Output of the PERMANOVA analysis carried out on microbiome composition based on Bray Curtis dissimilarity. Data were computed in the R environment using the command “adonis” and “pairwise_adonis”. Df (degrees of freedom), SumOfSqs (sum of squares), sig (significance). P-value threshold: 0.05.

```
PERMANOVA 9999 permutations, Bray by Sponge Species

         Df SumOfSqs R2      pseudo.F p.value   sig
Species   4  19.9733 0.86167 82.538   9.999e-05 ***
Residual 53   3.2064 0.13833
Total    57  23.1797 1.00000
---

Pairwise comparisons:
                          pairs   F.Model        R2 p.value p.adjusted sig
1      C.crambe vs C.reniformis 1858.8265 0.9862056   0.001      0.001  **
2      C.crambe vs P.ficiformis  977.1121 0.9770026   0.001      0.001  **
3          C.crambe vs Seawater  328.7622 0.9453650   0.001      0.001  **
4          C.crambe vs C.nucula 1097.1283 0.9812186   0.001      0.001  **
5  C.reniformis vs P.ficiformis 2012.4820 0.9877300   0.001      0.001  **
6      C.reniformis vs Seawater 1038.0392 0.9801707   0.001      0.001  **
7      C.reniformis vs C.nucula 2666.1475 0.9914471   0.001      0.001  **
8      P.ficiformis vs Seawater  498.3502 0.9651399   0.001      0.001  **
9      P.ficiformis vs C.nucula 1344.6909 0.9853447   0.001      0.001  **
10         Seawater vs C.nucula  657.6557 0.9762490   0.001      0.001  **
```

Table S5. Output of the PERMANOVA analysis carried out on microbiome composition based on Jaccard index. Data were computed in the R environment using the command “adonis” and “pairwise adonis”. Df (degrees of freedom), SumOfSqs (sum of squares), sig (significance). P-value threshold: 0.05.

```
PERMANOVA 9999 permutations, Jaccard by Sponge species

         Df SumOfSqs R2      pseudo.F p.value   sig
Species   4   16.909 0.72452 34.849   9.999e-05 ***
Residual 53    6.429 0.27548
Total    57   23.338 1.00000
---

Pairwise comparisons:

                          pairs    F.Model        R2 p.value p.adjusted sig
1      C.crambe vs C.reniformis  655.40788 0.9618437   0.001      0.001  **
2      C.crambe vs P.ficiformis  292.99787 0.9272147   0.001      0.001  **
3          C.crambe vs Seawater   80.73459 0.8094944   0.001      0.001  **
4          C.crambe vs C.nucula  439.79865 0.9544270   0.001      0.001  **
5  C.reniformis vs P.ficiformis  977.87727 0.9750717   0.001      0.001  **
6      C.reniformis vs Seawater  836.04288 0.9754971   0.001      0.001  **
7      C.reniformis vs C.nucula 1370.78302 0.9834981   0.001      0.001  **
8      P.ficiformis vs Seawater  412.10684 0.9581499   0.001      0.001  **
9      P.ficiformis vs C.nucula  764.72797 0.9745135   0.001      0.001  **
10         Seawater vs C.nucula  706.51731 0.9778552   0.001      0.001  **
```

Table S6. Output of PERMANOVA analysis based on DEICODE robust Aitchison dissimilarity. Data were computed in the R environment using the command "adonis" and "pairwise_adonis". Df (degrees of freedom), SumOfSqs (sum of squares), MeanSqs (mean squares), sig (significance). P-value threshold: 0.05.

```
PERMANOVA 9999 permutations, DEICODE RPCA by Sponge Species

          Df SumsOfSqs MeanSqs pseudo.F      R2 p.value   sig
Species    3   130.007  43.336  313.14 0.95332 9.999e-05 ***
Residuals 46     6.366   0.138         0.04668
Total     49   136.373                 1.00000
---

Pairwise comparisons:

                         pairs  F.Model        R2 p.value p.adjusted sig
1     C.crambe vs C.reniformis 239.0073 0.9018895   0.001      0.001  **
2     C.crambe vs P.ficiformis 278.8018 0.9237911   0.001      0.001  **
3         C.crambe vs C.nucula 415.0320 0.9518384   0.001      0.001  **
4 C.reniformis vs P.ficiformis 204.2311 0.8909398   0.001      0.001  **
5     C.reniformis vs C.nucula 180.9373 0.8872202   0.001      0.001  **
6     P.ficiformis vs C.nucula 200.8119 0.9094252   0.001      0.001  **
```

Table S7. Output of the Welch's test for the log ratios of the DEICODE analysis. The DEICODE analysis refers to the Robust Aitchison Principal Component analysis performed to identify the most relevant ASVs differentiating the four sponge spcies, whose log-ratios were selected and investigated in the qiime2 environment, within the Qurro tool. The final statistics was carried out in the R environment. P-value threshold: 0.05.

```
Welch's Test for the Deicode Log ratios

-------------------------------------------------------------
  statistic  : 239.1966
  num df     : 3
  denom df   : 23.30919
  p.value    : 1.216199e-17

-------------------------------------------------------------
Pairwise comparisons:

     Level (a)    Level (b)      p.value   No difference
1     C.crambe     C.nucula 8.794012e-10          Reject
2     C.crambe C.reniformis 1.728541e-12          Reject
3     C.crambe P.ficiformis 9.070778e-11          Reject
4     C.nucula C.reniformis 5.219573e-12          Reject
5     C.nucula P.ficiformis 9.075164e-05          Reject
6 C.reniformis P.ficiformis 5.215286e-11          Reject
```

Table S8. Microbiome dataset. The table is reported in the excel file Table_S8.xlsx. SampleID refers to the label of each sample. Abundance is the relative abundance of each ASV in each sample. Sponge species refers to the contracted scientific name of each sponge species: *Crambe crambe*, *Petrosia ficiformis*, *Chondrosia reniformis*, *Chondrilla nucula*. Order refers to the best annotated taxonomic rank used to perform this analysis.

Table S9. Output of PERMANOVA analysis carried out on the FAPROTAX putative functions. Data were computed in the R environment using the command "adonis" and "pairwise_adonis". Df (degrees of freedom), SumOfSqs (sum of squares), MeanSqs (mean squares), sig (significance). P-value threshold: 0.05.

```
PERMANOVA 9999 permutations, putative functions by Sponge Species

          Df SumsOfSqs MeanSqs pseudo.F  R2     p.value   sig
Species    3    8.4393 2.81311  80.408 0.83693 9.999e-05 ***
Residuals 47    1.6443 0.03499         0.16307
Total     50   10.0836                 1.00000
---

Pairwise comparisons:
                         pairs   F.Model        R2 p.value p.adjusted sig
1 P.ficiformis vs C.reniformis  13.44448 0.3408456   0.001      0.001  **
2     P.ficiformis vs C.crambe  98.33154 0.8038118   0.001      0.001  **
3     P.ficiformis vs C.nucula 120.30687 0.8513873   0.001      0.001  **
4     C.reniformis vs C.crambe 102.97376 0.7984086   0.001      0.001  **
5     C.reniformis vs C.nucula 110.00777 0.8270778   0.001      0.001  **
6         C.crambe vs C.nucula  44.80676 0.6808838   0.001      0.001  **
```

Table S10. Metabolomics dataset. The table is reported in the excel file Table_S10.xlsx. ID refers to the unique identifier of each chemical feature. Sample refers to the original label assigned to each sample. Abundance refers to the relative abundance of each chemical feature within each sample. Site refers to the code of each sampling sites: SA (Sant'Anna), GF (Grotta Mago external). Species refers to the contracted scientific name of the sponge species used in this study: *Crambe crambe, Petrosia ficiformis, Chondrosia reniformis, Chondrilla nucula*. SampleID refers to the sample identifier used for the chemical analyses. Molecular classes refer to the molecular class identified by Lipidsearch for each molecular feature.

Table S11. Output of PERMANOVA analysis carried out on metabolomics dataset. Data were computed in the R environment using the command “adonis” and “pairwise_adonis”. Df (degrees of freedom), SumOfSqs (sum of squares), MeanSqs (mean squares), sig (significance). P-value threshold: 0.05.

	Df	SumOfSqs	R2	F	Pr(>F)
	<dbl>	<dbl>	<dbl>	<dbl>	<dbl>
Sponge_Species	3	6939.802	0.6806528	23.44526	0.01
Residual	33	3256.002	0.3193472	*NA*	*NA*
Total	36	10195.803	1.0000000	*NA*	*NA*

Pairwise comparisons:	Df	SumsOfSqs	F.Model	R2	p.value	p.adjusted	sig
Crambe crambe vs Chondrilla nucula	1	3435.6355	24.342824	0.5888041	0.001	0.006	*
Crambe crambe vs Chondrosia reniformis	1	868.3485	9.897704	0.3679758	0.001	0.006	*
Crambe crambe vs Petrosia ficiformis	1	2029.2142	19.767230	0.5685592	0.001	0.006	*
Chondrilla nucula vs Chondrosia reniformis	1	3926.9468	41.187681	0.6958827	0.001	0.006	*
Chondrilla nucula vs Petrosia ficiformis	1	1380.4209	12.516906	0.4389293	0.002	0.012	.
Chondrosia reniformis vs Petrosia ficiformis	1	2061.8782	38.508341	0.7064669	0.001	0.006	*

Table S12. List of contracted and full names of the metabolite classes involved in this study.

**Metabolite class acronym**	**Metabolite class name**
AcCa	Acyl_Carnitine
AcHexStE	AcylGlcStigmasterol_Esters
Cer	Ceramides
CerP	Ceramides_phosphate
ChE	Cholesterol_Ester
CmE	Campesterol_Esters
Co	Coenzymes
DG	Diglycerides
Hex1Cer	Simple_Glc_series
Hex2Cer	Simple_Glc_series
Hex3Cer	Simple_Glc_series
LPC	LysoPhosphatidylcholines
LPE	LysoPhosphatidylethanolamines
LPG	LysoPhosphatidylglycerols
LPI	LysoPhosphatidylinositols
MG	Monoglycerides
MGDG	Monogalactosyldiacylglycerol
MePC	Methyl_Phosphatidylcholines
PC	Phosphatidylcholines
PE	Phosphatidylethanolamines
PG	Phosphatidylglycerols
PI	Phosphatidylinositols
PIP	Phosphatidylinositols
PMe	Phosphatidylmethanols
PS	Phosphatidylserines
SM	Sphingomyelins
SPH	Sphingosines
ST	Sulfatides
SiE	Sitosterol_Esters

StE	Stigmasterol_Esters
TG	Triglycerides
WE	Wax_Esters
ZyE	Zymosterol_Esters

Supplementary references

1. Herlemann, D. P. *et al.* Transitions in bacterial communities along the 2000 km salinity gradient of the Baltic Sea. *ISME J* **5**, 1571–1579 (2011).
2. Martinez Arbizu, P. pairwiseAdonis: Pairwise multilevel comparison using adonis. *R package version 0.4* (2020).
3. Bastian, M., Heymann, S. & Jacomy, M. Gephi: an open source software for exploring and manipulating networks. *Third international AAAI conference on weblogs and social media* (2009) doi:10.1136/qshc.2004.010033.
4. K. R. Clarke. Non-parametric multivariate analyses of changes in community structure. *Australian Journal of Ecology* (1993).
5. Anderson, M. J., Gorley, R. N. & Clarke, K. R. PERMANOVA+ for PRIMER: Guide to Software and Statistical Methods. in *Plymouth, UK* (2008).
