# Supplementary material for: High microbiome and metabolome diversification in coexisting sponges with different bio-ecological traits
Source: Commun Biol. 2024 Apr 8;7:422. doi: 10.1038/s42003-024-06109-5 (PMC11001883; doi:10.1038/s42003-024-06109-5)
Supplement: Supplementary file 3 — Description of Additional Supplementary Files [file 42003_2024_6109_MOESM3_ESM.pdf]

Description of Additional Supplementary Files

File name: Supplementary Data 1
Description: Microbiome dataset.

File name: Supplementary Data 2
Description: Metabolomics dataset.
