# Supplementary material for: High microbiome and metabolome diversification in coexisting sponges with different bio-ecological traits
Source: Commun Biol. 2024 Apr 8;7:422. doi: 10.1038/s42003-024-06109-5 (PMC11001883; doi:10.1038/s42003-024-06109-5)
Supplement: Supplementary file 7 — Reporting Summary [file 42003_2024_6109_MOESM7_ESM.pdf]

nature portfolio

Corresponding author(s): Laura Núñez Pons

Last updated by author(s): Feb 23, 2024

Reporting Summary

Nature Portfolio wishes to improve the reproducibility of the work that we publish. This form provides structure for consistency and transparency in reporting. For further information on Nature Portfolio policies, see our Editorial Policies and the Editorial Policy Checklist.

Statistics

For all statistical analyses, confirm that the following items are present in the figure legend, table legend, main text, or Methods section.

n/a	Confirmed	
☐	☒	The exact sample size (*n*) for each experimental group/condition, given as a discrete number and unit of measurement
☒	☐	A statement on whether measurements were taken from distinct samples or whether the same sample was measured repeatedly
☐	☒	The statistical test(s) used AND whether they are one- or two-sided<br>*Only common tests should be described solely by name; describe more complex techniques in the Methods section.*
☒	☐	A description of all covariates tested
☒	☐	A description of any assumptions or corrections, such as tests of normality and adjustment for multiple comparisons
☒	☐	A full description of the statistical parameters including central tendency (e.g. means) or other basic estimates (e.g. regression coefficient) AND variation (e.g. standard deviation) or associated estimates of uncertainty (e.g. confidence intervals)
☐	☒	For null hypothesis testing, the test statistic (e.g. *F*, *t*, *r*) with confidence intervals, effect sizes, degrees of freedom and *P* value noted<br>*Give P values as exact values whenever suitable.*
☒	☐	For Bayesian analysis, information on the choice of priors and Markov chain Monte Carlo settings
☒	☐	For hierarchical and complex designs, identification of the appropriate level for tests and full reporting of outcomes
☒	☐	Estimates of effect sizes (e.g. Cohen's *d*, Pearson's *r*), indicating how they were calculated

Our web collection on statistics for biologists contains articles on many of the points above.

Software and code

Policy information about availability of computer code

Data collection	NO software was used for data collection
Data analysis	As desribed in the Methods and Suplementary sections: Diversity comparisons and differential abundance for compositional data were calculated in QIIME2 v.2019.10 and R Studio (v1.2.5033) was used to perform statistical analyses and to generate plots with specific packages (Qiime2R, Phyloseq, Vegan, Ggplot2 R packages). LipidSearch v4.0 (Thermo Fisher Scientific) and Metaboanalyst v4.0 (https://www.metaboanalyst.ca/) were used for metabolomics data analysis. Similarity percentage analysis (SIMPER) was run on PRIMER 6+. FAPROTAX v1.2.4 was used for predicted functions annotations.

For manuscripts utilizing custom algorithms or software that are central to the research but not yet described in published literature, software must be made available to editors and reviewers. We strongly encourage code deposition in a community repository (e.g. GitHub). See the Nature Portfolio guidelines for submitting code & software for further information.

Data

Policy information about availability of data

All manuscripts must include a data availability statement. This statement should provide the following information, where applicable:

- Accession codes, unique identifiers, or web links for publicly available datasets
- A description of any restrictions on data availability
- For clinical datasets or third party data, please ensure that the statement adheres to our policy

Sequencing data for this study can be found under the Bioproject ID PRJNA851757. The metagenomic reads for the 16S V3-V4 region are available at NCBI under the accession numbers SAMN29249576-SAMN29249593, SAMN29249594-SAMN29249614, SAMN29249648-SAMN29249669. Metabolomics data have been deposited to the EMBL-EBI MetaboLights database (DOI: 10.1093/nar/gkz1019, PMID:31691833) with the identifier MTBLS5125. The complete dataset can be accessed here: https://www.ebi.ac.uk/metabolights/MTBLS5125.

Human research participants

Policy information about studies involving human research participants and Sex and Gender in Research.

Reporting on sex and gender	*Use the terms sex (biological attribute) and gender (shaped by social and cultural circumstances) carefully in order to avoid confusing both terms. Indicate if findings apply to only one sex or gender; describe whether sex and gender were considered in study design whether sex and/or gender was determined based on self-reporting or assigned and methods used. Provide in the source data disaggregated sex and gender data where this information has been collected, and consent has been obtained for sharing of individual-level data; provide overall numbers in this Reporting Summary. Please state if this information has not been collected. Report sex- and gender-based analyses where performed, justify reasons for lack of sex- and gender-based analysis.*
Population characteristics	*Describe the covariate-relevant population characteristics of the human research participants (e.g. age, genotypic information, past and current diagnosis and treatment categories). If you filled out the behavioural & social sciences study design questions and have nothing to add here, write "See above."*
Recruitment	*Describe how participants were recruited. Outline any potential self-selection bias or other biases that may be present and how these are likely to impact results.*
Ethics oversight	*Identify the organization(s) that approved the study protocol.*

Note that full information on the approval of the study protocol must also be provided in the manuscript.

Field-specific reporting

Please select the one below that is the best fit for your research. If you are not sure, read the appropriate sections before making your selection.

☐ Life sciences ☐ Behavioural & social sciences ☒ Ecological, evolutionary & environmental sciences

For a reference copy of the document with all sections, see nature.com/documents/nr-reporting-summary-flat.pdf

Ecological, evolutionary & environmental sciences study design

All studies must disclose on these points even when the disclosure is negative.

Study description	We integrated microbiome characterization, correlated with metabolomic patterns and microbial predicted functions profiling of four sponge species –Petrosia ficiformis, Chondrosia reniformis, Crambe crambe and Chondrilla nucula– coexisting in the same geographic area of the Mediterranean Sea. We used five replicates from each species coming from three sites with similar conditions, which were grouped in the same sampling group by sponge species (after checking their within-group homogeneity), so yielding 15 individuals per species for the comparison. We further compared microbial communities of the surrounding seawater with the sponge microbiomes. Seawater samples were collected in triplicate from each site, and similarly grouped after checking their homogeneity.
Research sample	Our samples were individuals of marine samples, Phylum Porifera, Class Demospongiae, appertaning to four different species: Petrosia ficiformis, Chondrosia reniformis, Crambe crambe and Chondrilla nucula. They were wild sponge individuals living on rocks, some of massive or incrusting growth.
Sampling strategy	NA Sample size for sponge individuals was chosen according to or exceeding standards in the field, which is often 3 (we applied n = 5 from 3 replicated sites that were in the end n = 15 individuals). Seawater samples were collected in triplicate from each site.
Data collection	Microbiome data was collected from MISeq sequencing after matagenomic DNA extarctios, and metabolomics data was collected from LC-MS from the corresponding chemical extacts.

Timing and spatial scale	All sponge samples were collected within 3 days, this is between the 23th and the 25th of April 2018, due to fieldwork constriccions getting all the collections in the same day. This represents no relevant spacing that could affect the variables measured, as the conditions were similar. As for the sequencing and metabolomics analyses, both were perfomed in one run on either approach.
Data exclusions	NO data were excluded from the study.
Reproducibility	For means of reproducibility, in the microbiome characterization we always used the same amount of clean sponge tissue, rinsed with sterile seawater, and we extracted the DNA the same day using a kit and following manufacturers instructions, further performing steps on randomized batches of tubes. As for the metabolomics, we always used the same amount of liofilized sponge tissue, and ran extractions with the same procedures, and LC-MS in the randomized positions in the same run. Wild individuals were selected in the same area separated by at least 10 m.
Randomization	Organisms from the same species were allocated in the same group, as the study comapares sponge microbiomes and metabolomes between the different species, being hence "Species" the diving co-variate.
Blinding	Blinding was not really a relevant feature in our study as we appled uniform techniques and analyses across all the dataset, also hardly samples could be distinguished within the same group, due ti the nature of the samples (i.e., sponge pieces)..

Did the study involve field work? ☒ Yes ☐ No

Field work, collection and transport

Field conditions	In our sampling campaigns, days were for the most part a bit cloudy and water temperature was 18ºC, with good visibility.
Location	Sponge samples of four target species were collected along with ambient seawater (n = 3 per site) in spring 2018 by scuba diving at 2-5 m depth around the island of Ischia, (Southern Tyrrhenian Sea, Mediterranean Sea, Italy), in three sampling sites where all species were highly abundant [62,63]: Castello Aragonese (CCO; 40°43'55.9"N - 13°57'52.9"E), Grotta Mago external (GF; 40° 42'41.6"N - 13°57'51.4"E) and Sant'Anna (SA; 40°43'36.5"N - 13°57'43.4"E).
Access & import/export	We accessed the collecting sites at Ischia Island with Stazione Zoologica Anton Dohrn boats and scuba diving service, located at Ischia Marine Station. We hence required no major importation or exportation procedures of samples, as they were brought right at the wet lab on the island. The sites were accessed with local autorization from the Area Marina Protetta Regno di Nettuno (Ischia).
Disturbance	Sampling of sponge pieces was perfomed applying a clean cut with a knife on the sponge donor, plus during the collection procedure, we always were careful to leave a wild piece of the donor sponge on site to preserve its life.

Reporting for specific materials, systems and methods

We require information from authors about some types of materials, experimental systems and methods used in many studies. Here, indicate whether each material, system or method listed is relevant to your study. If you are not sure if a list item applies to your research, read the appropriate section before selecting a response.

Materials & experimental systems

n/a	Involved in the study	
☒	☐	Antibodies
☒	☐	Eukaryotic cell lines
☒	☐	Palaeontology and archaeology
☐	☒	Animals and other organisms
☒	☐	Clinical data
☒	☐	Dual use research of concern

Methods

n/a	Involved in the study	
☒	☐	ChIP-seq
☒	☐	Flow cytometry
☒	☐	MRI-based neuroimaging

Animals and other research organisms

Policy information about studies involving animals; ARRIVE guidelines recommended for reporting animal research, and Sex and Gender in Research

Laboratory animals	The study did not involve laboratory animals.
Wild animals	Pieces of ~10 cm3 were colected from wild donor marine sponges, always leaving a viable piece on site. Sponges were collected by scuba diving in individual plastic zip bags and brought to the lab to be immediately processed for sample preservations for the diverse analytical approaches (snap frozen and kept at -80ºC, frozen at -20ºC).

Reporting on sex	This information has not been collected, as it is not relevant to the study.
Field-collected samples	Wild sponge sample pieces collected individual plastic zip bags were brought to the lab to be immediately processed for sample preservations for the diverse analytical approaches (snap frozen and kept at -80ºC, frozen at -20ºC).
Ethics oversight	NO ethical appoval was required as our target organisms are not protected species, and are of clonal growth. In this sense, in the collection procedure, we always were careful to leave a wild piece of the donor sponge on site to preserve its life.

Note that full information on the approval of the study protocol must also be provided in the manuscript.
